# Excimer light effect on neurogenic inflammation in active versus stable psoriasis lesions

**DOI:** 10.1007/s10103-024-04005-2

**Published:** 2024-02-01

**Authors:** Marwa S. El-Mesidy, Yomna A. Metwally, Zeinab A. Nour, Maha F. Elmasry

**Affiliations:** 1 Dermatology Department, Kasr Al Ainy Faculty of Medicine, Cairo University, Cairo, Egypt; 2 Biochemistry Department, Kasr Al Ainy Faculty of Medicine, Cairo University, Cairo, Egypt

**Keywords:** Psoriasis, Excimer light, Neurogenic inflammation, Substance P

## Abstract

Neurogenic inflammation, mediated by T helper 17 cell (Th17) and neurons that release neuropeptides such as substance P (SP), is thought to play a role in the pathogenesis of psoriasis. Excimer light is used in the treatment of psoriasis via induction of T cell apoptosis. The objective of this study is to study the effect of excimer light on active versus stable psoriasis and investigate the levels of substance P and its receptor in both groups. The study included 27 stable and 27 active psoriatic patients as well as 10 matched healthy controls. Clinical examination (in the form of local psoriasis severity index (PSI) and visual analogue scale (VAS)) was done to determine disease severity, level of itching, and quality of life. Tissue levels of SP and neurokinin-1 receptor (NK-1R) were measured by ELISA before and after 9 excimer light sessions in 43 patients. A statistically significant lower levels of PSI and VAS were reached after therapy with no significant difference between the stable and active groups. The mean tissue levels of SP before therapy were significantly higher than the control group. Lower levels of SP and NK-1 receptor were found after treatment overall and in each group. Excimer therapy can be effective for both stable and active plaque psoriasis and this effect could be partly through its role on ameliorating the neurogenic inflammation.

## Introduction

Psoriasis shows an altered expression of various neuropeptides and their receptors [1] such as substance P (SP), histamine, serotonin, calcitonin gene–related peptide, adenosine, glucagon like peptide, and somatostatin [2].

SP is secreted by nerves and inflammatory cells and acts by binding to the neurokinin-1 receptor (NK-1 R) having a proinflammatory effect in psoriasis [2] and stimulates chemotaxis of lymphocytes and neutrophils [3] and promotes T helper 17 (Th17) to produce interleukin-17 (IL-17) [4].

Neuropeptides regulate transducing nerve impulses to signals read by the immune cells thus exacerbating the inflammatory reaction. The enhancement of neurotrophin expression in psoriasis supports this phenomenon.

SP also affects the vascular and cellular inflammatory components. It translocates P-selectin to endothelial cell membranes and enhances the expression of E-selectin thus intensifying the inflammation. Moreover, it helps in the migration and adhesion of leukocytes to endothelial cells. It binds to NK-1R on different cells and this linkage between neuropeptides and immune cells plays a role in modulating the inflammation [5].

Treatment of psoriasis includes topical treatments, systemic therapy, phototherapy, biologics, and immune modulators. However, it is usually challenging due to the side effects encountered, resistance to treatment, high expenses, and variable responses encountered. By addressing these challenges, therapy may be optimized for everyone improving their lives [6].

Excimer light decreased cytokine expression in psoriasis and led to clinical remission and so could be an effective treatment for psoriasis [7].

It induces apoptosis in keratinocytes and T lymphocytes [8] as well as reduces the numbers of pathogenic memory/effector T cells infiltrating lesional epidermis and dermis [9] and promotes the degeneration of dorsal root nerve fibers proving its effect on neurogenic inflammation [10].

The aim of this work was to examine the effect of excimer light on active versus stable psoriasis plaques to detect the ideal patient whose life may benefit more from this form of therapy. Also, it aimed at investigating the levels of substance P and its receptor before and after the excimer light sessions, in a trial to detect its effect on neurogenic inflammation.

## Patients and methods

The study was conducted on 54 psoriasis patients and 10 healthy controls, during the time interval from May 2022 to October 2022 in a university hospital after approval by the Research Ethical Committee (REC) with code MS-66–2022 on 17–4-2022. Written informed consents were obtained from all participants.

Included patients were plaque psoriasis patients, more than 18 years old, of both genders, and with psoriasis affecting less than 10% of the body surface area. Patients were divided into two groups: 27 patients in each group. The stable group included patients with psoriasis lesions for three or more months. The active group had developing psoriasis lesions arising within 1 month, enlarging plaques, annular lesions, or any lesion arising at sites of trauma within 10–20 days^.^

History of physical trauma preceding the lesion within 10–20 days was recorded, and itching assessment was done by visual analogue scale [11] in addition to the 4-item itch questionnaire [12]. Assessments of psoriasis area and severity index (PASI) [13] and psoriasis severity index for the single targeted plaque (PSI) [14] as well as digital photography were done. Tissue levels of human SP and NK-1 R were measured by enzyme-linked immunosorbent assay technique (ELISA) in 43 patients.

A 3-mm punch skin biopsy was taken from the edge of a psoriatic plaque of 43 patients and from 10 healthy controls under local anesthesia using Lidocaine®. The skin biopsies were stored at – 20 °C in Eppendorf tubes with 900 µl phosphate buffer saline (PBS) added to each Eppendorf tube. Tissue samples with PBS were homogenized by a grinder, then centrifuged for 5 min at 10,000 revolutions per minute (rpm) speed. The supernatant was removed to be stored at − 80 °C till use for measuring the levels of human substance P and neurokinin-1 R by ELISA. Kits were supplied by ELK Biotech Co., LTD (China) (Human Substance P Catalogue Number ELK1039 and Human Neurokinin-1 R Catalogue Number ELK1512).

### Admission for excimer light

Regarding the used excimer light, Excilite-µ handpiece, *MEL@308 nm* (DEKA, Italy), was used in the study. It has a monochromatic source able to emit a non-coherent light with a narrow band centered on 308 nm in the UVB. The emission is a continuous wave of 1–90 s and is uniform on the area where the treatment can take place, 30 cm^2^ at maximum (5 × 6 cm) with power density 50 mW/cm^2.^

Two sessions were performed weekly for a total of 9 sessions. The starting dose ranged from 300 to 900 mJ/cm^2^ according to the skin type and induration of the plaque. For an induration score of 1, the initial dose was 300–400 mJ/cm^2^ according to the site and skin type; for a score of 2: 500–600 mJ/cm^2^; and for a score of 3: 700–900 mJ/cm^2^. If mild or no erythema occurred, dose was increased by 30%. If moderate erythema, warmth, or skin sensitivity occurred, dose was increased by 20%. Then, the dose was further increased by 10–20% until discomfort prevented further increase, followed by dose maintenance. In case of blistering, treatment was postponed until resolution and subsequent dose was reduced by 20% (PHAROS EX-308 Operations Guide)^5^. One day after the last session, itching and PSI as well as levels of SP and NK-1 R were reassessed. Then, patients were followed up clinically for 1 and 3 months.

### Statistical methods

Data were coded and entered using the Statistical Package for the Social Sciences (SPSS) version 28 (IBM Corp., Armonk, NY, USA). Data were summarized using mean, standard deviation, median, minimum, and maximum in quantitative data and using frequency (count) and relative frequency (percentage) for categorical data. Comparisons between quantitative variables were done using the non-parametric Kruskal–Wallis and Mann–Whitney tests. For comparison of serial measurements within each patient, the non-parametric Friedman test and Wilcoxon signed-rank test were used.

For comparing categorical data, chi-square (*χ*^2^) test was performed. Exact test was used instead when the expected frequency was less than 5. Correlations between quantitative variables were done using Spearman correlation coefficient. ROC curve was constructed with area under curve analysis performed to detect the best cutoff value of substance P and receptor level for detection of cases. *P* values less than 0.05 were considered statistically significant.

## Results

Fifty-four patients were enrolled in the study as well as ten controls. Patients and controls were matched homogenously regarding age, sex, and skin type (*P* > 0.05). Demographic and clinical data of patients and controls are shown in Table 1.

**Table 1:** Demographic and clinical data of patients and controls

	Variable	Patients *n* = 54	Controls *n* = 10
Age	Range	19–66	19–42
	Mean ± SD	33.33 ± 14.34	30.1 ± 8.76
Sex (No. (%))	Males	24 (44.4%)	5 (50%)
	Females	30 (55.6%)	5 (50%)
Skin type (No. (%))	Type III	10 (18.5%)	1 (10%)
	Type IV	38 (70.4%)	7 (70%)
	Type V	6 (11.1%)	2(20%)
Activity (No. (%))	Stable	27 (50%)	
	Active	27 (50%)	
Stress (No. (%))	Yes	38 (70.4%)	
	No	16 (29.6%)	
Treatments other than topicals (No. (%))	Yes	15 (27.8%)	
	No	39 (72.2%)	
Treatments other than topicals details (No. (%))	Narrow band UVB	2 (3.7%)	
	Methotrexate	7 (13%)	
	Acitretin	2 (3.7%)	
	Methotrexate and narrow band UVB and acitretin	2 (3.7)	
	Yes	2 (3.7%)	
	No	39 (72.2%)	
Trauma (No. (%))	Yes	10 (18.5%)	
	No	44 (81.5%)	

*n* number, *SD* standard deviation; *P value < 0.05 is significant

Twenty-seven patients (50%) were active psoriasis patients and the other twenty-seven (50%) were stable psoriasis patients. The duration of disease among patients ranged from 0.4 to 180 months with a mean of 46.08 ± 49.32 months. The duration of disease ranged from 0.96 to 120 months in the stable group and from 0.48 to 180 months in the active group, with no statistically significant difference between the 2 groups (*P* > 0.05).

The body surface area (BSA) affected by psoriasis ranged from 0.5 to 7% with a mean of 3.30 ± 1.93%. The BSA ranged from 0.5 to 5% in the stable group with a mean of 2.37 ± 1.49 and from 1 to 7% with a mean of 4.22 ± 1.89 in the active group, with a statistically significant difference between the 2 groups (*P* < 0.001), with more affected body surface area in the active group. PASI score ranged from 2 to 11 with a mean of 5.54 ± 1.85.

**Fig. 1:**
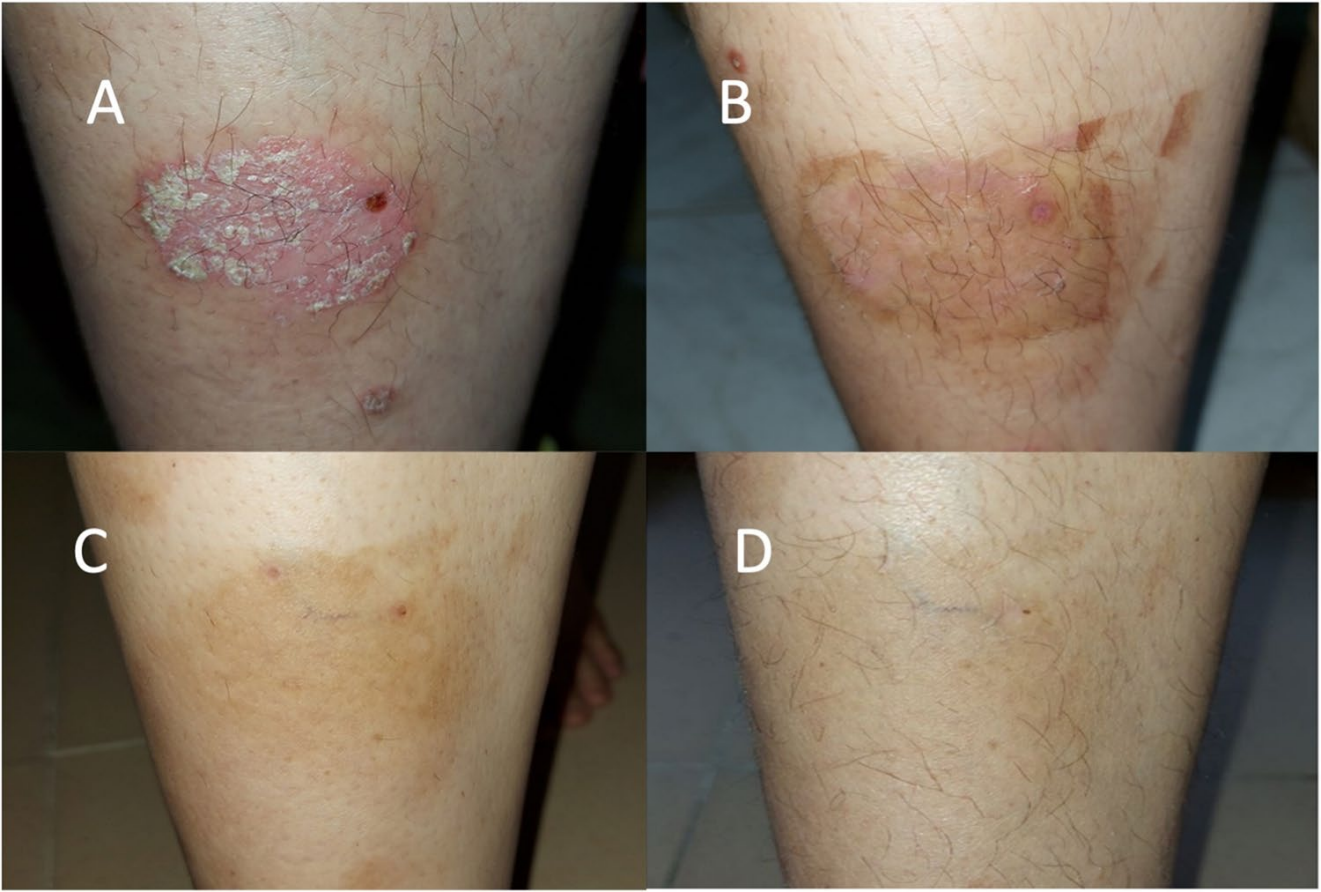
**A** A 34-year-old female with active psoriatic plaque on the right leg (progressively enlarging) before treatment (PSI 5). **B** One day after 9 sessions showing marked improvement apart from post-inflammatory hyperpigmentation (PSI 1). **C** After 1 month showing less post-inflammatory hyperpigmentation (PSI 0). **D** After 3 months showing almost normal skin (PSI 0)

**Fig. 2:**
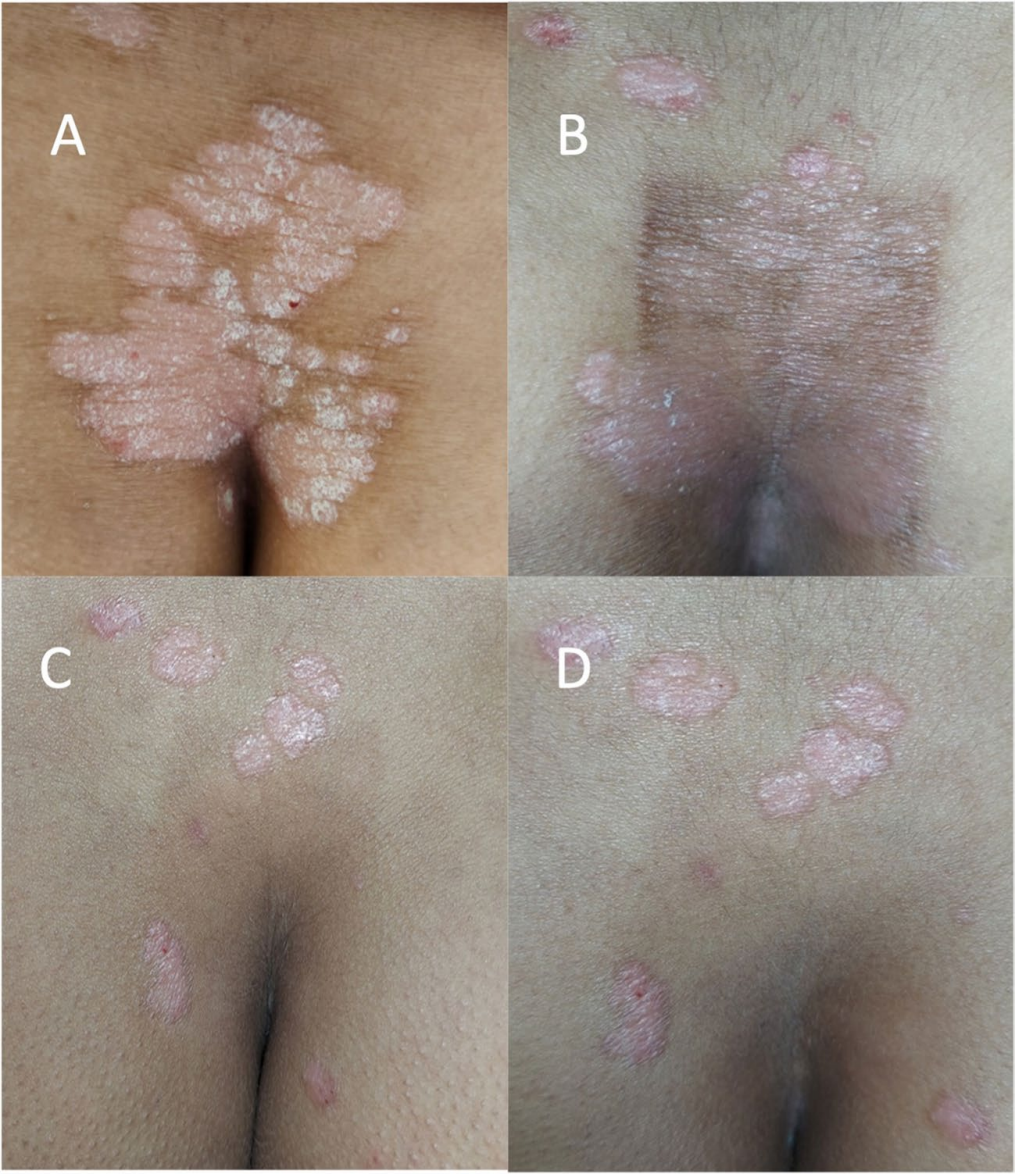
**A** A 45-year-old female with stable psoriatic plaque on the lower back before treatment (PSI 5). **B** One day after 9 sessions showing improvement and post-inflammatory hyperpigmentation (PSI 2). **C** After 1 month showing less post-inflammatory hyperpigmentation and new lesions around the treated area (PSI 2). **D** After 3 months showing similar presentation to that of 1 month (PSI 2)

PSI score ranged from 2 to 11 with a mean of 5.54 ± 1.85 with no statistically significant difference between the 2 groups (*P* > 0.05). VAS score ranged from 2 to 10 with a mean of 6.69 ± 2.22. Comparisons between the results of both groups with statistical significance are shown in Table 2 and Figs. 1 and 2.

**Table 2:** Comparison between stable and active groups regarding clinical scores before and after treatment

	Stable group			Active group			*P* value
	Mean ± SD	Median	Range	Mean ± SD	Median	Range	
PSI before	5.11 ± 1.80	5.00	2.00–9.00	5.63 ± 2.11	5.00	2.00–11.00	0.400
PSI after	2.11 ± 1.89	2.00	0.00–6.00	2.19 ± 1.62	2.00	0.00–6.00	0.745
*P* value	< 0.001			< 0.001			
VAS before	6.56 ± 2.10	7.00	2.00–10.00	6.81 ± 2.37	6.00	3.00–10.00	0.694
VAS after	2.70 ± 2.09	2.00	0.00–9.00	3.22 ± 2.82	2.00	0.00–9.00	0.674
*P* value	< 0.001			< 0.001			
4-item before	9.67 ± 3.68	9.00	3.00–15.00	9.63 ± 3.09	9.00	3.00–16.00	0.827
4-item after	5.30 ± 3.53	5.00	0.00–16.00	5.37 ± 3.63	6.00	0.00–16.00	0.752
*P* value	< 0.001			< 0.001			
Erythema before	1.85 ± 0.46	2.00	1.00–3.00	2.07 ± 0.68	2.00	1.00–3.00	0.171
Erythema after	1.00 ± 0.83	1.00	0.00–3.00	1.07 ± 0.62	1.00	0.00–2.00	0.600
*P* value	< 0.001			< 0.001			
Induration before	1.56 ± 0.85	2.00	0.00–3.00	1.70 ± 0.99	2.00	0.00–4.00	0.620
Induration after	0.48 ± 0.64	0.00	0.00–2.00	0.52 ± 0.75	0.00	0.00–2.00	0.976
*P* value	< 0.001			< 0.001			
Scaling before	1.70 ± 0.78	2.00	0.00–3.00	1.85 ± 0.82	2.00	1.00–4.00	0.621
Scaling after	0.63 ± 0.74	0.00	0.00–2.00	0.59 ± 0.57	1.00	0.00–2.00	0.946
*P* value	< 0.001			< 0.001			
PSI% change	− 56.59 ± 43.42	− 60.00	− 100.00–100.00	− 56.04 ± 39.29	-60.00	− 100.00–50.00	0.965
VAS% change	− 57.15 ± 28.97	− 60.00	− 100.00–0.00	− 51.46 ± 37.13	-57.14	− 100.00–0.00	0.639
Item% change	− 38.88 ± 37.00	− 28.57	− 100.00–60.00	− 38.71 ± 38.48	− 28.57	− 100.00–60.00	0.842

*P* value > 0.05 is non-significant. *PSI* psoriasis severity index, *VAS* visual analogue scale

**Fig. 3:**
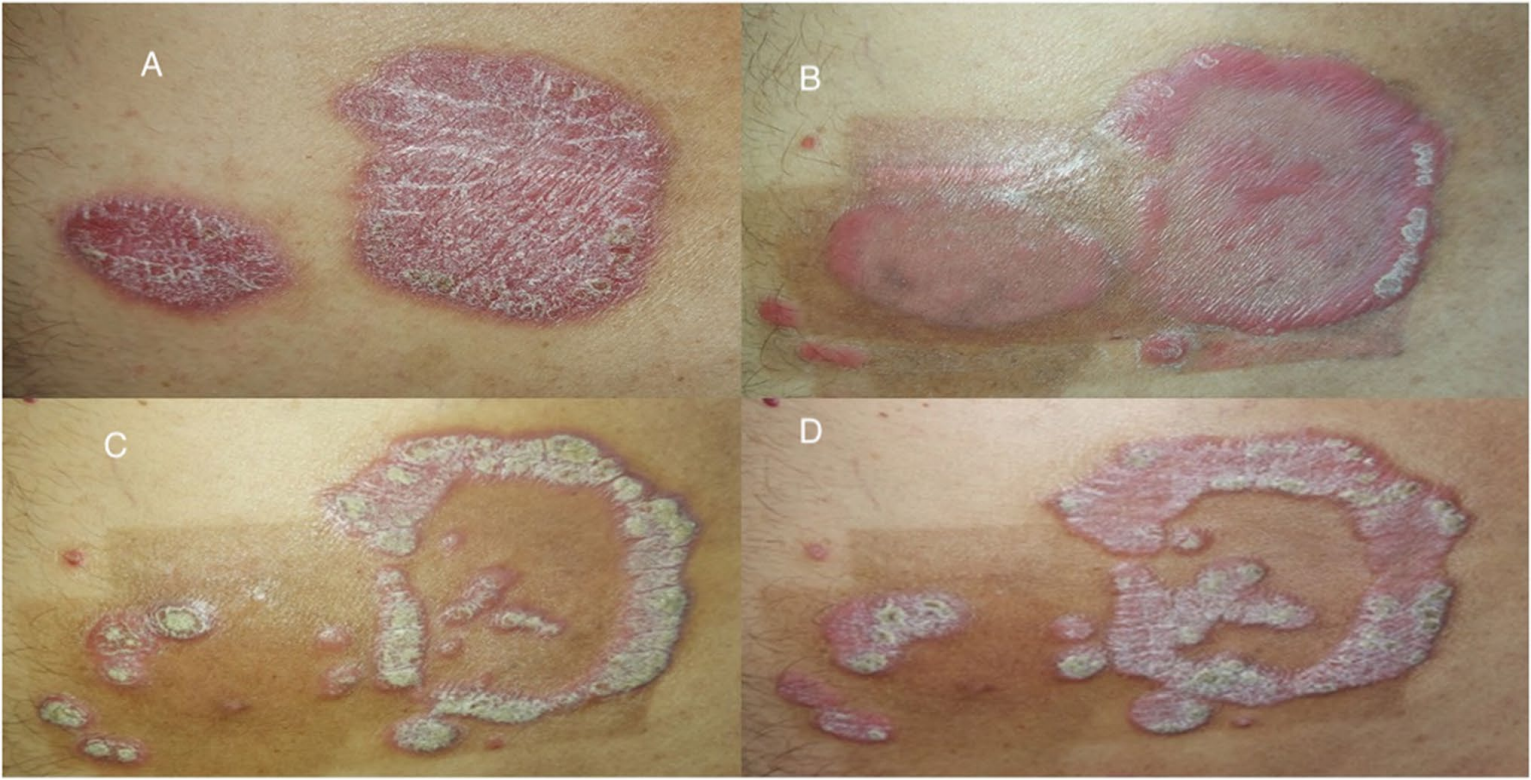
Remaining annular edge. **A** Before treatment. **B** One day after 9 sessions showing annularity that persisted during the follow-up at 1 month (**C**) and 3 months (**D**)

Thirteen patients (24.1%) showed a remaining annular edge of the psoriatic plaque after treatment: 3 (11.1%) stable patients and 10 (37%) active patients with a statistically significant difference between the 2 groups (*P* < 0.05) favoring the active group (Fig. 3).

At 1 month follow-up, PSI scores ranged from 0 to 8 with a mean of 2.02 ± 2.33. PSI scores ranged from 0 to 8 with a mean of 1.76 ± 2.31 in the stable group and from 0 to 8 with a mean of 2.3 ± 2.36 in the active group with no statistically significant difference between the 2 groups (*P* > 0.05).

At 3 months follow-up, PSI scores ranged from 0 to 8 with a mean of 2.28 ± 2.39. PSI scores ranged from 0 to 7 with a mean of 2.42 ± 2.24 in the stable group and from 0 to 8 with a mean of 2.14 ± 2.59 in the active group with no statistically significant difference between the 2 groups (*P* > 0.05).

Upon comparing PSI scores after treatment and 1 month later, there was no statistically significant difference (*P* > 0.05) overall or in each group (*P* > 0.05) and no statistically significant difference between the 2 groups (*P* > 0.05). Upon comparing PSI scores after treatment and 3 months later, there was no statistically significant difference.

Erythema occurred in 26 patients (48.14%): 15 stable (55.5%) and 11 active (40.7%) patients with no statistically significant difference between the 2 groups (*P* > 0.05). Blistering occurred in 4 patients (7.4%): 2 stable (7.4%) patients and 2 active (7.4%) patients with no statistically significant difference between the 2 groups (*P* > 0.05). Post-inflammatory hyperpigmentation occurred in 36 patients (66.6%): 17 stable (62.9%) and 19 active (70.3%) patients with no statistically significant difference between the 2 groups (*P* > 0.05).

Increased size of the targeted plaque occurred in 7 patients (12.96%): 4 stable (14.8%) and 3 active (11.1%) patients with no statistically significant difference between the 2 groups (*P* > 0.05). Eruption of new plaques far from the treated area was noticed in 5 patients (9.2%): 3 stable (11.1%) and 2 active (7.4%) patients with no statistically significant difference between the 2 groups (*P* > 0.05).

### Biochemical assessment

Biochemical assessment was done on 43 patients (22 stable and 21 active) in comparison to 10 controls (elaborated in Table 3). Confidence interval of SP tissue level ranged from 0.75 to 0.96, cutoff level at 67.77 ng/ml with 86% sensitivity and 100% specificity, while confidence interval of NK-1 receptor tissue level ranged from 0.43 to 0.72. Biochemical assessment in all patients before and after treatment is shown in Table 4.

**Table 3:** Comparison of SP and NK-1 receptor tissue level before treatment between patients and controls

	Cases			Controls			*P* value
	Mean ± SD	Median	Range	Mean ± SD	Median	Range	
Conc SP before ng/ml	116.89 ± 96.07	83.56	51.77–521.61	66.42 ± 0.53	66.55	65.50–67.10	** < 0.001**
Conc R before pg/ml	18.40 ± 23.42	3.24	0.74–58.50	2.56 ± 0.49	2.55	1.89–3.24	0.426
Conc SP after ng/ml	82.17 ± 31.28	72.80	52.58–181.81	66.42 ± 0.53	66.55	65.50–67.10	0.065
Conc R after pg/ml	6.21 ± 13.94	1.99	0.64–56.50	2.56 ± 0.49	2.55	1.89–3.24	0.342

Value in bold indicates highly significant result

*Conc *concentration;* P *value < 0.001 is highly significant

**Table 4:** Comparison of SP and NK-1 receptor levels before and after treatment in all patients

	Cases			*P* value
	Mean ± SD	Median	Range	
Conc SP before ng/ml	116.89 ± 96.07	83.56	51.77–521.61	**0.004**
Conc SP after ng/ml	82.17 ± 31.28	72.80	52.58–181.81	
Optic density SP before	0.14 ± 0.11	0.11	0.07–0.74	**0.004**
Optic density SP after	0.11 ± 0.03	0.10	0.07–0.20	
Conc R before pg/ml	18.40 ± 23.42	3.24	0.74–58.50	**0.026**
Conc R after pg/ml	6.21 ± 13.94	1.99	0.64–56.50	
Optic density R before	0.96 ± 0.28	0.90	0.54–1.70	**0.019**
Optic density R after	0.81 ± 0.20	0.80	0.50–1.40	

Values in bold indicate significant results

* *P* value < 0.05 is significant; *Conc* concentration

There was no statistically significant difference between the active and stable groups regarding substance P (SP) tissue level either before or after treatment (*P* > 0.05). Also, there was no statistically significant difference between the 2 groups regarding neurokinin-1 (NK-1 R) tissue level either before or after treatment (*P* > 0.05).

## Discussion

Excimer devices are used for treatment of psoriasis [14] usually in patients having a suboptimal response to topical treatments [15]. They are indicated for treatment of mild, moderate, or severe localized psoriasis [14]. It is particularly beneficial in patients having a suboptimal response or contraindications to topical or intralesional treatments [15]. Excimer therapy has also proven efficacy in treating difficult psoriasis subtypes such as scalp, nail, and palmoplantar psoriasis [14].

The results of the current study come in agreement with Feldman et al. who conducted excimer laser sessions on stable mild to moderate plaque psoriasis**,** where 72% of patients achieved at least 75% clearing in an average of 6.2 treatments [16]. Another study performed excimer laser therapy on 26 patients with chronic psoriasis, where PSI score improved > 50% in 90% of patients [17]. Better achieved results could be due to the use of laser not light.

Also, in agreement with the current study, one study conducted 10 excimer light sessions on severe (PSI ≥ 6) localized chronic plaque psoriasis in Japanese patients where 83% improvement in PSI was reached [18].

Maximum improvement was less than the current study, which could be related to the different ethnicity, skin type, or the higher severity.

The explanation of itching decrease could be attributed to the decrease in nerve fibers following phototherapy [19].

In the current study, clinical improvement in the active psoriasis group was comparable to that in the stable group. Most side effects occurred in the lighter skin type (III). This indicates that excimer light is both effective and safe in active localized plaque psoriasis, with caution in lighter skin types.

Remaining annular edge could be explained by the difference of physical properties between lasers and lamps. Light devices do not produce a strict monochromatic wavelength [20] and is also divergent, which means that the periphery of lesion receives less intensity than the center [21]. Another explanation could be due to the activity in the margin that expands peripherally, which explains why it remains resistant to therapy.

Side effects in the active group (such as erythema, blistering, increased size of the targeted plaque, or appearance of new plaques) were less than the stable group, but post-inflammatory hyperpigmentation was more in the active group, with no statistically significant difference in the 2 groups regarding side effects. Most side effects (apart from hyperpigmentation) occurred in the lighter skin type (III). This indicates that excimer light is both effective and safe in active localized plaque psoriasis, with caution in lighter skin types.

Neurogenic inflammation, mediated by nociceptive neurons and T helper 17 cell (Th17) responses, plays a major role in the immune pathogenesis of psoriasis [3]. Nerve fibers release various types of neuropeptides that act on receptors on cells in different layers of the skin [2].

Effect on neurogenic inflammation was investigated in this study. The mean tissue levels of SP in the current study were significantly higher than the control group (*P* < 0.001) denoting its role in psoriasis pathogenesis.

This comes in accordance with Chan et al. who found out that SP-positive nerve fibers are more abundant in psoriatic plaques [22]. Other studies as well reported that SP in mature psoriatic lesions was greater than in healthy controls which indicates its role in the maintenance of psoriatic lesions [23].

However, other studies denied a difference in the number of SP-positive nerve fibers between lesional and non-lesional psoriatic skin. They reported that SP expression in immune cells, *not nerve fibers*, is increased in lesional skin [24].

In our study, there was no statistically significant difference between the stable and active groups regarding SP tissue level either before or after treatment, though levels were lower in the active group. Similarly, Nour et al. reported that nerve endings are diminished in acutely inflamed psoriatic lesions explained by the scratching in acutely inflamed lesions that lead to destruction of cutaneous nerve fibers and depletion of SP [25].

On the contrary, Pergolizzi et al. reported that protein gene product 9.5 (PGP 9.5)–positive nerve endings are decreased in mature psoriatic lesions and even not detected in long-term psoriasis. They concluded that neuropeptides would thus be low in chronic lesions compared to newer ones. They attributed the degeneration of nerve fibers in chronic lesions to the persistent inflammation [26].

Further studies are essential to detect the level of neuropeptides chronologically during the evolution of the psoriatic plaque.

Also, there was no statistically significant difference between the stable and active groups regarding NK-1 receptor tissue level either before or after treatment. In one study, NK-1 levels were found *insignificantly* higher in the exacerbation than the remission phase [27].

Lower levels of SP and NK-1 receptor were found after treatment, overall and in the stable and active group (but insignificantly in the active group), suggesting the normalizing effect of excimer light on SP and hence on improving pruritus in psoriasis patients. The insignificant decrease in SP and NK-1 receptor levels in the active group could suggest a higher degree of neurogenic inflammation or a higher reactivity of immune cells to SP during the active phase.

Kamo et al. proved that excimer lamp irradiation to nerve fibers in atopic mice decreases the density of both intraepidermal and dermal nerve fibers [10].

The insignificant decrease in SP and NK-1 receptor levels in the active group could suggest a higher degree of neurogenic inflammation or a higher reactivity of immune cells to SP during the active phase.

In conclusion, excimer therapy can be effective and safe for both stable and active plaque psoriasis, considering the skin type, that lesions are localized, the patient does not demonstrate a history of photosensitive psoriasis, and the physician’s assessment of each case. In addition, SP is significantly overexpressed in psoriatic lesions indicating its role in psoriasis pathogenesis via its effect on T helper 17 differentiation and IL-17 release. Suppression of neurogenic inflammation via downregulation of SP may represent an additional mechanism through which excimer therapy exerts its effect.

## Data Availability

The datasets generated during and/or analyzed during the current study are available from the corresponding author on reasonable request.
